# Characterization of Two Antimicrobial Peptides from Antarctic Fishes (*Notothenia coriiceps* and *Parachaenichthys charcoti*)

**DOI:** 10.1371/journal.pone.0170821

**Published:** 2017-01-25

**Authors:** Seung Chul Shin, In Hye Ahn, Do Hwan Ahn, Yung Mi Lee, Jungeun Lee, Jun Hyuck Lee, Han-Woo Kim, Hyun Park

**Affiliations:** 1 Unit of Polar Genomics, Korea Polar Research Institute (KOPRI), Incheon, Republic of Korea; 2 Department of Polar Sciences, Korea University of Science and Technology, Incheon, Republic of Korea; Seconda Universita degli Studi di Napoli, ITALY

## Abstract

We identified two antimicrobial peptides (AMPs) with similarity to moronecidin in Antarctic fishes. The characteristics of both AMPs were determined using moronecidin as a control. Moronecidin, which was first isolated from hybrid striped bass, is highly salt-resistant, and possesses broad-spectrum activity against various microbes. The moronecidin-like peptide from *Notothenia coriiceps* exhibited a narrower spectrum of activity and a higher salt sensitivity than moronecidin. The AMP from *Parachaenichthys charcoti* exhibited similar antimicrobial activity to moronecidin, and similar salt sensitivity. In an experiment to identify toxic effects, both of the moronecidin-like peptides from the Antarctic fishes exhibited lower hemolytic activity than moronecidin. In spite of its low toxicity, the AMP from *N*. *coriiceps* is unlikely to be considered as a candidate for antibiotic development, owing to its narrow spectrum of activity and high salt sensitivity. In contrast, the high salt resistance and broad-spectrum activity of the AMP from *P*. *charcoti* could be more advantageous for clinical use than moronecidin, since it could kill bacteria under physiological conditions with low toxicity. A further comparison of these two AMPs from Antarctic fishes with other AMPs could help to reduce the toxicity of AMPs for clinical use.

## Introduction

Antimicrobial agents have defeated many infectious diseases and have improved public health significantly. However, many pathogenic microorganisms are becoming resistant to several antimicrobial agents/drugs, and demand for novel antibiotics continues to grow [1].

Antimicrobial peptides (AMPs) may be one of the new generation of antibiotics to meet this demand [2, 3]. AMPs are crucial effector molecules of the innate immune response, present in most living organisms [4]. AMPs possess broad-spectrum antimicrobial activities against bacteria, fungi, and viruses [5]. Certain AMPs can kill pathogens that are resistant to almost all conventional antibiotics [6]. AMPs kill microorganisms using diverse mechanisms. AMPs can disrupt membrane structure by forming transmembrane pores, inhibiting cell-wall synthesis, and by inhibiting cytoplasmic membrane septum formation. Certain AMPs can inhibit enzymes and can inhibit the synthesis of proteins and nucleic acids [3, 7, 8]. However, AMPs also have drawbacks; these include instability, hemolytic activity, salt sensitivity, toxicity toward eukaryotic cells, susceptibility to proteolysis, and a higher cost of production compared with conventional antibiotics [2, 9, 10].

In spite of their drawbacks, some AMPs from the pool of thousands of natural peptides have been developed and validated as therapeutic agents [9–11]. The costs could be decreased by commercial-scale production by the pharmaceutical industry [12–14]. Indeed, several AMPs have proceeded to clinical trials [9–11]. However, the Food and Drug Administration (FDA) of the United States of America has not yet granted approval for the clinical use of any AMP.

Fish are frequently exposed to a wide variety of pathogens. Consequently, they are a good source for the discovery of new AMPs [15, 16]. Pardaxin [17], misgurin [18], cathelicidins [19, 20], defensins [21], NK-lysin [22], hepcidin [23], and piscidin [24–26] have been reported as AMPs in fish. These AMPs have been isolated from fish skin, gills, and intestines, or have been annotated in fish genomes. In Antarctic fishes, a piscidin-like AMP was isolated from the icefish *Chionodraco hamatus* [27]. Piscidin has potent, broad-spectrum activity against microorganisms [24–26]. Moronecidin, which is a member of the piscidin family of AMPs, was first isolated from the skin and gills of hybrid striped bass [24, 26]. Moronecidin is a 22-residue amphipathic alpha-helical peptide, which is C-terminally amidated. It exhibits broad-spectrum antimicrobial activity with low toxicity and high salt tolerance [24].

In this study, we discovered two moronecidin-like peptides in other Antarctic fishes (*Notothenia coriiceps* and *Parachaenichthys charcoti*). Using synthetic mature peptides, we investigated the spectrum of these AMPs, the effect of salt concentration on their activity, and their toxicity. To investigate whether these AMPs had distinct characteristics arising from their origin in fish that live in a cold environment, we tested the effect of temperature on their activity.

## Materials and Methods

### Molecular characterization of moronecidin-like peptides from Antarctic fishes

The amino acid sequence of a moronecidin-like peptide from *N*. *coriiceps* was obtained (NCBI reference sequence: XP_010768425.1). The cDNA sequence of a moronecidin-like peptide from *P*. *charcoti* was obtained from the assembled contigs, generated from mRNA-seq in the liver (GenBank accession number: KX344030). Theoretical isoelectric point (pI) values, net charges, and molecular weights (MW) were predicted using the *Peptide Property Calculator* from Innovagen (http://pepcalc.com/ppc.php). Schiffer-Edmundson wheel representations of AMPs were obtained using *HeliQuest* [28] from the ExPASy website (http://expasy.org/tools/). Homologous AMP sequences were obtained from the NCBI database and were aligned using *ClustalW* (http://www.genome.jp/tools/clustalw/). A phylogenetic tree was constructed by the neighbor-joining method using the *Mega 5* program, on full-length amino acid sequences [29].

### Synthetic peptides

Putative mature AMPs from Antarctic fishes, bearing C-terminal amidation, were synthesized commercially to 95% purity (Peptron, Republic of Korea) (Table 1). The synthesized peptides were purified by high-performance liquid chromatography (Shimadzu, Kyoto, Japan) on a Shiseido Capcell Pak C18 column (Shiseido, Co., Ltd., Tokyo, Japan). Molecular weights of the synthesized peptides were confirmed using liquid chromatography/mass spectrometry (Agilent, CA, USA). Immediately prior to use, each peptide was reconstituted to 100 μM in phosphate-buffered saline (pH 7.4).

**Table 1:** Synthetic peptides.

Name	Sequence	Abbreviation	Net charge at pH 7	pI	Calculated MW (measured MW)
***Hybrid striped bass***	FFHHIFRGIVHVGKTIHKLVTG-NH_2_	moro-NH_2_	4.4	14	2543.0 (2542.4)
***Notothenia coriiceps***	FFWHHIGHALDAAKRVHGMLSG-NH_2_	moroNC-NH_2_	2.4	11.39	2486.9 (2486.2)
***Parachaenichthys charcoti***	FFGHLFRGIINVGKHIHGLLSG-NH_2_	moroPC-NH_2_	3.3	14	2418.8 (2418.0)

### Microbial strains and culture conditions

The microbial strains used in this study are listed in Table 2. These include Gram-negative and Gram-positive bacteria, filamentous fungi, and yeast species. All microbial isolates were obtained from the Korean Collection for Type Cultures (KCTC), the American Type Culture Collection (ATCC), or the Polar and Alpine Microbial Collection (PAMC) of the Korea Polar Research Institute. For liquid cultures, tryptic soy broth (BD Diagnostic Systems, Sparks, MD, USA) and nutrient broth (BD Diagnostic Systems) were used. The culture medium and temperature used for each strain is listed in Table 2.

**Table 2:** Microbial strains and culture conditions.

Name	Microbial type	Culture broth	Growth temperature (°C)
*Pseudomonas aeruginosa* (ATCC 15442)	Gram-negative bacterium	Tryptic soy broth	30
*Burkholderia cepacia* (ATCC 25416)	Gram-negative bacterium	Nutrient broth	30
*Enterobacter cloacae* (ATCC 13047)	Gram-negative bacterium	Nutrient broth	30
*Shigella sonnei* (ATCC 29930)	Gram-negative bacterium	Nutrient broth	30
*Psychrobacter* sp. (PAMC 21119)	Gram-negative bacterium	Nutrient broth	25
*Psychrobacter* sp. (PAMC 25501)	Gram-negative bacterium	Nutrient broth	25
*Escherichia coli* DH5α	Gram-negative bacterium	Nutrient broth	30
*Flavobacteria* sp. (PAMC 22217)	Gram-negative bacterium	Nutrient broth	15
*Lacinutrix algicola* (AKS293^T^)	Gram-negative bacterium	Nutrient broth	15
*Enterococcus faecalis* (ATCC 29212)	Gram-positive bacterium	Tryptic soy broth	30
*Streptococcus pyogenes* (ATCC 19615)	Gram-positive bacterium	Tryptic soy broth	30
*Staphylococcus aureus* (ATCC 33591)	Gram-positive bacterium	Nutrient broth	30
*Listeria monocytogenes* (ATCC 15313)	Gram-positive bacterium	Nutrient broth	30
*Aspergillus fumigatus* (ATCC 26430)	Filamentous fungus	Nutrient broth	30
*Candida glabrata* (ATCC 2001)	Yeast	Nutrient broth	30
*Candida tropicalis* (ATCC 20115)	Yeast	Nutrient broth	30

### Antimicrobial activity assay

The minimal inhibitory concentration (MIC) was determined as described previously [30]. To determine the MIC, a microbial culture was incubated with an AMP for 18 h in 96-well plate (Bioneer, Republic of Korea). Each well contained 90 μL of a microbial cell suspension at 1 × 10^5^ cfu/mL, and 10 μL of a particular AMP that had been serially diluted in growth medium. MICs were defined as the lowest peptide concentrations that inhibited microbial growth completely. To evaluate the effect of temperature on the MICs, microbial cultures in the presence of AMPs were incubated for 24 h below 25°C, since microbial growth is slowed at low temperatures. Under different temperature conditions, microbial strains were cultured with AMPs for 18 h. *E*. *coli* DH5α was cultured between 20–37°C, and *Psychrobacter* spp. PAMC 25501 and PAMC 21119 were cultured between 5–25°C. The effect of cations was tested on AMPs by culturing *Psychrobacter* sp. PAMC 25501 at 20°C for 24 h to determine the MIC in modified LB medium with either no salt or various concentrations of NaCl (0–1000 mM), MgCl_2_ (0–40 mM), or CaCl_2_ (0–20 mM).

### Hemolytic activity assay

The hemolytic activity of AMPs was determined against sheep blood and horse blood (Oxoid Ltd., London, United Kingdom) [24]. Freshly packed sheep and horse erythrocytes (1 mL) were washed with phosphate-buffered saline (pH 7.4). AMPs were added to 90 μL of a 1% erythrocyte suspension (1:10 dilution of washed erythrocytes) in microcentrifuge tubes. The samples were incubated for 30 min at 37°C, and then centrifuged for 10 min at 4000 rpm at room temperature. The supernatants were transferred carefully to a 96-well plate, and the optical density was determined at 405 nm. The percentage of hemolysis was defined relative to the hemolysis obtained by treating the erythrocyte suspension with 0.1% SDS (100% hemolysis).

## Results and Discussion

### Genes encoding moronecidin-like peptides in antarctic fishes

Piscidin, which is a cationic peptide comprising 22-amino acids, has broad-spectrum antimicrobial activity against Gram-positive and Gram-negative bacteria [24]. It is also known to induce apoptosis in cancer cells [31]. To detect piscidin homologs in Antarctic fishes, we investigated the genome of *N*. *coriiceps* using the *BlastP* tool, and we identified a gene encoding a moronecidin-like peptide with NCBI accession number XP_010768425.1. The gene encoding a moronecidin-like peptide in *N*. *coriiceps* consists of 4 exons, which encode 77 amino acids. Since we had assembled contigs using RNA-seq data generated from the liver tissue of *P*. *charcoti*, we were also able to identify the cDNA sequence encoding a 79 amino acid moronecidin-like peptide among those contigs.

### Phylogeny and molecular evolution of piscidins

**Fig 1:**
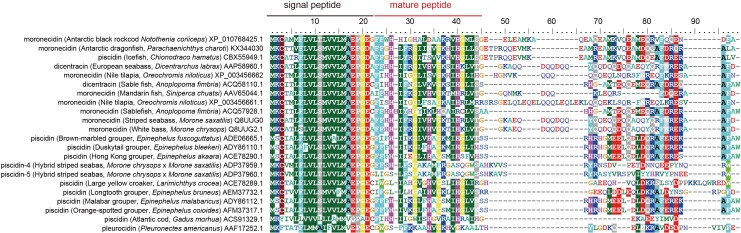
Alignment of the two novel moronecidin-like peptides with other known piscine AMPs. The signal peptides and mature peptides of AMPs are well conserved in fish species. Accession number: dicentracin (European seabass, *Dicentrarchus labrax*) AAP58960.1; moronecidin (Nile tilapia, *Oreochromis niloticus*) XP_003456662; dicentracin (Sablefish, *Anoplopoma fimbria*) ACQ58110.1; moronecidin (Mandarin fish, *Siniperca chuatsi*) AAV65044.1; moronecidin (Nile tilapia, *Oreochromis niloticus*) XP_003456661.1; moronecidin (Sablefish, *Anoplopoma fimbria*) ACQ57928.1; moronecidin (Striped seabass, *Morone saxatilis*) Q8UUG0; moronecidin (White bass, *Morone chrysops*) Q8UUG2.1; piscidin (Brown-marbled grouper, *Epinephelus fuscoguttatus*) ADE06665.1; piscidin (Duskytail grouper, *Epinephelus bleekeri*) ADY86110.1; piscidin (Hong Kong grouper, *Epinephelus akaara*) ACE78290.1; piscidin (Hybrid striped seabass, *Morone chrysops* × *Morone saxatilis*) ADP37959.1; piscidin (Hybrid striped seabass, *Morone chrysops* × *Morone saxatilis*) ADP37960.1; piscidin (Large yellow croaker, *Larimichthys crocea*) ACE78289.1; piscidin (Longtooth grouper, *Epinephelus bruneus*) AEM37732.1; piscidin (Malabar grouper, *Epinephelus malabaricus*) ADY86112.1; piscidin (Orange-spotted grouper, *Epinephelus coioides*) AFM37317.1; moronecidin (Antarctic black rockcod, *Notothenia coriiceps*) XP_010768425.1; moronecidin (Antarctic dragonfish, *Parachaenichthys charcoti*) KX344030; pleurocidin (*Pleuronectes americanus*) AAF17252.1; piscidin (Icefish, *Chionodraco hamatus*) CBX55949.1; piscidin (Atlantic cod, *Gadus morhua*) ACS91329.1.

**Fig 2:**
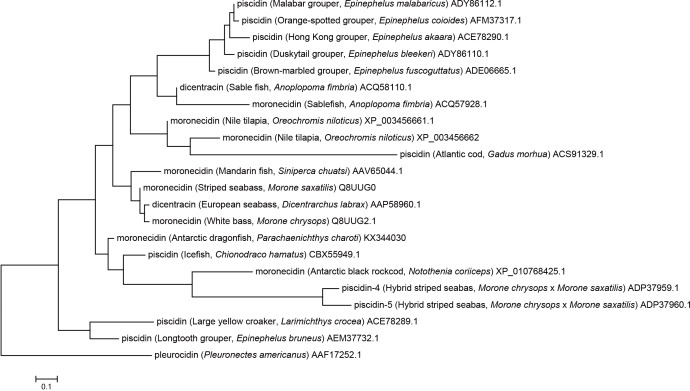
Phylogenetic tree showing the relationship between the two moronecidin-like peptides from Antarctic fishes and other known AMPs. The accession number of each AMP is the same as that given in Fig 1. The phylogenetic tree was constructed by the neighbor-joining method using the bootstrap test with 2000 replicates. The scale bar denotes a 0.1 change per amino acid position.

The amino acid sequence of the newly identified AMP from *P*. *charcoti* shares 64% identity with that from hybrid striped bass, and 78% identity with that from *Chionodraco hamatus*. For *N*. *coriiceps*, the amino acid sequence of its AMP has comparatively low identity (43%) with moronecidin from hybrid striped bass. The AMP from *P*. *charcoti* has 43% identity with the AMP from *N*. *coriiceps*. An alignment with homologous AMPs shows that both the signal peptide and mature peptide are well conserved in the two novel moronecidin-like peptides (Fig 1). A phylogenetic analysis was also constructed, based on the amino acid sequences of other known piscine AMPs. The AMP from *P*. *charcoti* is closely related to the AMP from *C*. *hamatus*. In contrast, the AMP from *N*. *coriiceps* is more closely related to piscidin-4 and piscidin-5 from hybrid striped bass (Fig 2). All three AMPs from Antarctic fishes are located in single clade, together with piscidin-4 and -5 from hybrid striped bass.

### Secondary structures of the AMPs

**Fig 3:**
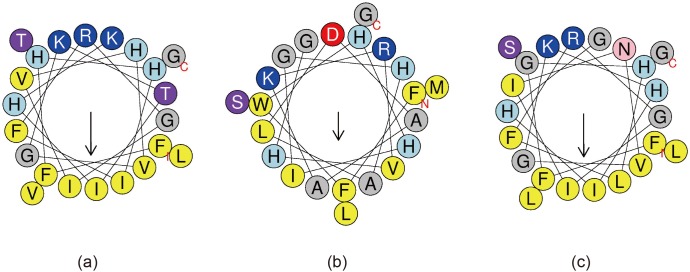
**Helical wheel diagram depicting amphipathic alpha-helical conformations of (a) moro (b) moroNC, and (c) moroPC.** Hydrophobic residues are yellow, positively charged residues are blue, and negatively charged residues are red. Particular polar residues are violet (threonine and serine), pink (asparagine and glutamine) or sky blue (histidine). The arrows represent the helical hydrophobic moment.

Schiffer-Edmundson helical wheel modeling of mature peptides from Antarctic fishes shows amphipathic alpha-helix conformations, in which hydrophobic and hydrophilic residues are on opposite sides of the alpha-helix (Fig 3). The mature moronecidin (moro) from hybrid striped bass and the mature peptide from *P*. *charcoti* (moroPC) each have a higher hydrophobicity score (0.627 and 0.617, respectively) than the mature peptide from *N*. *coriiceps* (moroNC; hydrophobicity score: 0.449). The hydrophobic moment, which is a measure of the amphiphilicity of a helix, was represented using *HeliQuest* [28]. The hydrophobic moments of moro, moroPC, and moroNC are 0.556, 0.559, and 0.365, respectively. Of the three peptides, moroNC has the lowest amphiphilicity.

### Antimicrobial activity

Moronecidin from hybrid striped bass displayed broad-spectrum antibacterial activity. Consequently, we determined the antimicrobial activity using synthetic, amidated AMPs (moro-NH_2_, moroNC-NH_2_, and moroPC-NH_2_) bearing the amino acid sequences found in Antarctic fishes (Table 3). The AMPs from Antarctic fishes showed strong activity against *Shigella sonnei*, *Psychrobacter* sp., and *Escherichia coli* DH5α (MIC < 12.5 μM), but did not exhibit antibacterial activity (up to 50 μM) against the Gram-negative bacteria *Pseudomonas aeruginosa* or *Burkholderia cepacia* (Table 3). Only moro-NH_2_ showed activity against *Pseudomonas aeruginosa* at 50 μM. Synthetic AMPs from hybrid striped bass and *P*. *charcoti* exhibited antibacterial activity against *Enterobacter cloacae* above 25 μM. In the case of Gram-positive bacteria, *Enterococcus faecalis*, *Streptococcus pyogenes*, *Staphylococcus aureus* and *Listeria monocytogenes* were sensitive to both AMPs from the Antarctic fishes below 25 μM, with the exception of moroNC-NH_2_ against *E*. *faecalis*. All three of the AMPs had antimicrobial activity against *Candida tropicalis*. A similar spectrum of activity and MIC against bacteria was seen for moroPC-NH_2_ and moronecidin from hybrid striped bass. In contrast, moroNC-NH_2_ exhibited weaker or non-existent antimicrobial activity against certain species. A relatively low hydrophobicity and pI value for moroNC-NH_2_ might account for its narrower spectrum and lower antibacterial activity. Nonetheless, for particular bacterial strains, moroNC-NH_2_ has similar antibacterial activity as the other two peptides, in spite of these differences in its physicochemical properties.

**Table 3:** Antimicrobial spectrum of synthetic, amidated moronecidin-like peptides from Antarctic fishes.

		AMP (μM)	
	moro-NH_2_	moroNC-NH_2_	moroPC-NH_2_
***Gram-negative bacteria***			
*Pseudomonas aeruginosa* (ATCC 15442)	50	>50	>50
*Burkholderia cepacia* (ATCC 25416)	>50	>50	>50
*Enterobacter cloacae* (ATCC 13047)	25	>50	25
*Shigella sonnei* (ATCC 29930)	5	12.5	5
*Psychrobacter* sp. (PAMC 25501)	2.5	5	2.5
*E*. *coli* DH5α	5	12.5	5
***Gram-positive bacteria***			
*Enterococcus faecalis* (ATCC 29212)	5	>50	25
*Streptococcus pyogenes* (ATCC 19615)	2.5	25	2.5
*Staphylococcus aureus* (ATCC 33591)	2.5	25	5
*Listeria monocytogenes* (ATCC 15313)	2.5	12.5	5
***Yeast***			
*Candida glabrata* (ATCC 2001)	>50	>50	>50
*Candida tropicalis* (ATCC 20115)	5	5	5

Since Antarctic fishes live in a cold environment, below 2°C [32], and since AMPs from icefish have been shown to kill bacteria in a temperature-dependent manner [27], we evaluated the effect of temperature on the activities of moro-NH_2_, moroNC-NH_2_, and moroPC-NH_2_ against certain bacteria. *Psychrobacter* sp. PAMC 25501, isolated from Ny-Ålesund in Svalbard, Norway, was used to test activity up to ~25°C. *E*. *coli* DH5α was used to test activity from 15°C to 37°C (Tables 4 and 5). However, the activities of AMPs from hybrid striped bass and Antarctic fishes were unaltered by these temperature changes. To assess whether the AMPs from Antarctic fishes could be effective as innate immunity molecules in a cold environment, we also measured their antibacterial activity against an additional cold-loving bacterium (Table 6). *Lacinutrix algicola* AKS293^T^ isolated from marine sediment in the Southern Ocean [33], and *Flavobacteria* sp. PAMC 22217 isolated from the Arctic Ocean, were selected, along with *Psychrobacter* sp. PAMC 21119, which was isolated from the Antarctic permafrost [34]. Although moro-NH_2_ exhibited a narrow spectrum of activity in Table 3, both AMPs from Antarctic fishes were active enough to kill these additional cold-loving bacteria.

**Table 4:** Effect of temperature on AMP activity against *Psychrobacter* sp. PAMC 25501.

*Psychrobacter* sp. (PAMC 25501)		AMP (μM)	
	moro-NH_2_	moroNC-NH_2_	moroPC-NH_2_
**5**°C	2.5	**2.5**	2.5
**15**°C	2.5	**2.5**	2.5
**25**°C	2.5	**5**	2.5

**Table 5:** Effect of temperature on AMP activity against *E. coli* DH5α.

*E*. *coli* DH5α		AMP (μM)	
	moro-NH_2_	moroNC-NH_2_	moroPC-NH_2_
**15**°C	5	12.5	5
**20**°C	5	12.5	5
**30**°C	5	12.5	5
**37**°C	5	12.5	5

**Table 6:** AMP activity against *Psychrobacter* sp. *PAMC 25501*, *Flavobacteria* sp. PAMC 22217, and *Lacinutrix algicola* AKS293^T^.

		AMP (μM)	
	moro-NH_2_	moroNC-NH_2_	moroPC-NH_2_
*Psychrobacter* sp. PAMC 21119	1.25	2.5	1.25
*Psychrobacter* sp. PAMC 25501	1.25	2.5	1.25
*Flavobacteria* sp. PAMC 22217	2.5	5	1.5
*Lacinutrix algicola* AKS293^T^	1.25	12.5	1.25

### Salt sensitivity

AMPs are initially attracted to microbial membranes by electrostatic interactions, prior to forming pores [35]. These electrostatic interactions can be disrupted by salts, inhibiting membrane disruption. To be certified for clinical use, AMPs must be active at physiological salt concentrations (150 mM NaCl, 3 mM CaCl_2_, and 2 mM MgCl_2_) [Blood Test Results—normal ranges (http://www.bloodbook.com/ranges.html)].

Consequently, we investigated how much the activity of AMPs was inhibited by various concentrations of salts. We identified that AMPs from *P*. *charcoti* and hybrid striped bass were active under the physiological salt concentrations found in human blood [Blood Test Results—normal ranges (http://www.bloodbook.com/ranges.html)]. However, a 5-fold increase and a 2-fold increase in MIC values was observed in the presence of 5 mM MgCl_2_, or 5 mM CaCl_2_, respectively (Table 7). The MIC value for moro-NH_2_ increased to 12.5 mM, or 25 mM, in the presence of 500 mM NaCl or 5 mM CaCl_2_, respectively. We could not determine MIC values in the presence of physiological concentrations of MgCl_2_.

**Table 7:** Effects of monovalent and divalent cations on AMP activity against *Psychrobacter* sp. *PAMC 25501*.

*Psychrobacter* sp. *PAMC 25501*			
		Moronecidin (μM)	
	moro-NH_2_	moroNC-NH_2_	moroPC-NH_2_
**Control**	2.5	5	2.5
***NaCl***			
*50*	2.5	**5**	2.5
*100*	2.5	**6.25–12.5**	2.5
*500*	2.5–5	**12.5**	2.5–5
*1000*	5	**25**	5
***MgCl***_***2***_			
***1***	*2*.*5*	25	2.5
*5*	*12*.*5*	>50	12.5
*10*	*12*.*5*	>50	12.5
*50*	>50	>50	>50
*100*	>50	>50	>50
***CaCl***_***2***_			
***1***	*2*.*5*	5	2.5
*5*	*5*	**25**	5
*10*	*6*.*25–12*.*5*	**50**	12.5
*50*	50	**>50**	50
*100*	50	**>50**	>50

### Hemolytic activity

**Fig 4:**
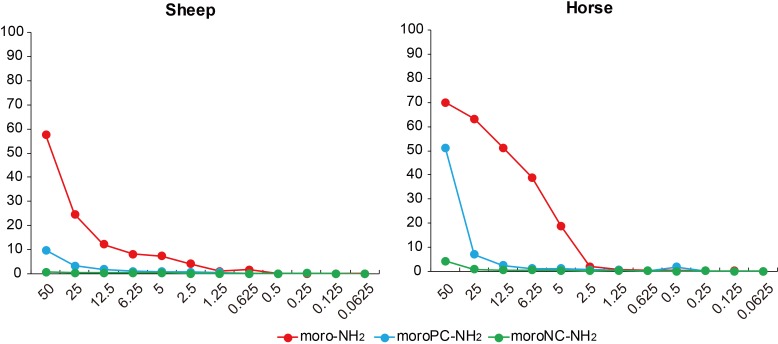
Hemolytic activities of moro-NH_2_, moroNC-NH_2_, and moroPC-NH_2._ The percentage of hemolysis was defined as the ratio of absorbance between the sample and an erythrocyte suspension treated with 0.1% SDS.

Since some types of AMP can lyse mammalian erythrocytes, hemolytic activity was tested as a therapeutic index. It is important to establish a low hemolytic activity for clinical use [2]. To investigate the possibility of using the AMPs from Antarctic fishes in a clinical setting, we used both sheep and horse erythrocytes to evaluate their hemolytic effects (Fig 4). Twelve concentrations were used for each AMP, and moro-NH_2_ was used as a control. Both AMPs from the Antarctic fishes caused a relatively lower percentage of hemolysis than moro-NH_2_. The hemolytic activity of moroNC-NH_2_ did not reach 10% with the highest concentration tested (50 μM). With 25 μM of an AMP from either of the Antarctic fishes, the hemolytic activity is below 10%. In contrast, moro-NH_2_ lysed up to 25% and 62% of the sheep and horse erythrocytes, respectively. moroNC-NH_2_ had a hemolytic activity below 1% at 25 μM, in both sheep and horse erythrocytes.

## Conclusions

The moronecidin-like peptide from *N*. *coriiceps* shows distinctive features for an AMP. The amino acid sequence has very low similarity with other AMPs. The most similar amino acid sequence is that of the piscidin-like antimicrobial peptide from the icefish, *C*. *hamatus*, with which it shares 55% identity. It shares 43% identity with moronecidin from hybrid striped bass. However, we could not find any advantages conferred by this distinctive amino acid sequence. In spite of its low toxicity, moroNC-NH_2_ has a narrow spectrum of antibacterial activity, and high salt sensitivity. These characteristics make it difficult to consider moroNC-NH_2_ for clinical use.

The moronecidin-like peptide from the Antarctic dragonfish, *P*. *charcoti*, is 88% identical to a piscidin-like antimicrobial peptide from *C*. *hamatus*, and has 64% identity with an AMP from hybrid striped bass. However, moroPC-NH_2_ produced similar results to moro-NH_2_ in experiments testing its salt sensitivity and its spectrum of activity against microbes. Furthermore, its toxicity was lower than that moro-NH_2_. At 12.5 μM of the AMPs tested, almost none of the sheep or horse erythrocytes were lysed. A characterized AMP from *C*. *hamatus* also has low hemolytic activity and broad-spectrum antimicrobial activity [27]. Although we could not test all of the AMPs from Antarctic fishes, AMPs from the species (*C*. *hamatus*, *N*. *coriiceps*, and *P*. *charcoti* AMPs) exhibit lower toxicity than moronecidin.

Currently, no AMPs have been approved as therapeutic agents by the FDA. Nonetheless, several cationic antimicrobial peptides (Pexiganan, Omiganan, Iseganan, and others) have entered into Phase III trials and have had their use clinically validated [9–11]. Moronecidin, which is a piscidin homolog, is also cationic antimicrobial peptide [36]. Therefore, in this study we investigated the characteristics of two moronecidin-like peptides from Antarctic fishes and tested whether those AMPs are suited for use as therapeutic agents. The AMP from *P*. *charcoti* exhibited high salt resistance, low toxicity, and broad-spectrum activity; these characteristics suggest that this AMP could be considered for inclusion in future clinical trials. We could not identify any temperature dependency for the activity of AMPs from *N*. *coriiceps* or *P*. *charcoti*; in contrast, the activity of the AMP from *C*. *hamatus* is known to be temperature-dependent [27]. We established that low toxicity appears to be a distinctive feature of AMPs from the Antarctic fishes studied to date. A further comparison of other AMPs, and AMPs from other Antarctic fishes, might facilitate the development of AMPs with lower toxicity [37].

## References

[1] Klein E, Smith DL, Laxminarayan R. Hospitalizations and deaths caused by methicillin-resistant Staphylococcus aureus, United States, 1999–2005. Emerg Infect Dis. 2007;13(12):1840–6. Epub 2008/02/09. PubMed Central PMCID: PMC2876761. 10.3201/eid1312.070629 18258033 PMC2876761

[2] Aoki W, Ueda M. Characterization of Antimicrobial Peptides toward the Development of Novel Antibiotics. Pharmaceuticals (Basel). 2013;6(8):1055–81. Epub 2013/11/28. PubMed Central PMCID: PMC3817730. 24276381 10.3390/ph6081055 PMC3817730

[3] Guilhelmelli F, Vilela N, Albuquerque P, Derengowski Lda S, Silva-Pereira I, Kyaw CM. Antibiotic development challenges: the various mechanisms of action of antimicrobial peptides and of bacterial resistance. Front Microbiol. 2013;4:353 Epub 2013/12/25. PubMed Central PMCID: PMC3856679. 10.3389/fmicb.2013.00353 24367355 PMC3856679

[4] Bowdish DM, Davidson DJ, Hancock RE. A re-evaluation of the role of host defence peptides in mammalian immunity. Curr Protein Pept Sci. 2005;6(1):35–51. Epub 2005/01/11. 15638767 10.2174/1389203053027494

[5] Hancock RE, Diamond G. The role of cationic antimicrobial peptides in innate host defences. Trends Microbiol. 2000;8(9):402–10. Epub 2000/09/16. 10989307 10.1016/s0966-842x(00)01823-0

[6] Park SC, Park Y, Hahm KS. The role of antimicrobial peptides in preventing multidrug-resistant bacterial infections and biofilm formation. Int J Mol Sci. 2011;12(9):5971–92. Epub 2011/10/22. PubMed Central PMCID: PMC3189763. 10.3390/ijms12095971 22016639 PMC3189763

[7] Brogden KA. Antimicrobial peptides: pore formers or metabolic inhibitors in bacteria? Nat Rev Microbiol. 2005;3(3):238–50. Epub 2005/02/11. 10.1038/nrmicro1098 15703760

[8] Teixeira V, Feio MJ, Bastos M. Role of lipids in the interaction of antimicrobial peptides with membranes. Prog Lipid Res. 2012;51(2):149–77. Epub 2012/01/17. 10.1016/j.plipres.2011.12.005 22245454

[9] Cézard C, Silva-Pires V, Mullié C, Sonnet P. Antibacterial peptides: a review. Science against Microbial Pathogens: Communicating Current Research and Technological Advances: Formatex Research Center. 2011.

[10] Gordon YJ, Romanowski EG, McDermott AM. A review of antimicrobial peptides and their therapeutic potential as anti-infective drugs. Current eye research. 2005;30(7):505–15. 10.1080/02713680590968637 16020284 PMC1497874

[11] Hancock RE. Cationic antimicrobial peptides: towards clinical applications. Expert opinion on investigational drugs. 2000;9(8):1723–9. 10.1517/13543784.9.8.1723 11060771

[12] Giuliani A, Pirri G, Nicoletto S. Antimicrobial peptides: an overview of a promising class of therapeutics. Open Life Sciences. 2007;2(1):1–33.

[13] Marr AK, Gooderham WJ, Hancock RE. Antibacterial peptides for therapeutic use: obstacles and realistic outlook. Current opinion in pharmacology. 2006;6(5):468–72. 10.1016/j.coph.2006.04.006 16890021

[14] Otvos L, Wade JD. Current challenges in peptide-based drug discovery. Frontiers in chemistry. 2014;2:62 10.3389/fchem.2014.00062 25152873 PMC4126357

[15] Rajanbabu V, Chen JY. Applications of antimicrobial peptides from fish and perspectives for the future. Peptides. 2011;32(2):415–20. Epub 2010/11/26. 10.1016/j.peptides.2010.11.005 21093512

[16] Noga EJ, Ullal AJ, Corrales J, Fernandes JM. Application of antimicrobial polypeptide host defenses to aquaculture: Exploitation of downregulation and upregulation responses. Comp Biochem Physiol Part D Genomics Proteomics. 2011;6(1):44–54. Epub 2010/06/30. 10.1016/j.cbd.2010.06.001 20584633

[17] Oren Z, Shai Y. A class of highly potent antibacterial peptides derived from pardaxin, a pore-forming peptide isolated from Moses sole fish Pardachirus marmoratus. Eur J Biochem. 1996;237(1):303–10. Epub 1996/04/01. 8620888 10.1111/j.1432-1033.1996.0303n.x

[18] Park CB, Lee JH, Park IY, Kim MS, Kim SC. A novel antimicrobial peptide from the loach, Misgurnus anguillicaudatus. FEBS Lett. 1997;411(2–3):173–8. Epub 1997/07/14. 9271200 10.1016/s0014-5793(97)00684-4

[19] Uzzell T, Stolzenberg ED, Shinnar AE, Zasloff M. Hagfish intestinal antimicrobial peptides are ancient cathelicidins. Peptides. 2003;24(11):1655–67. Epub 2004/03/17. 10.1016/j.peptides.2003.08.024 15019197

[20] Maier VH, Dorn KV, Gudmundsdottir BK, Gudmundsson GH. Characterisation of cathelicidin gene family members in divergent fish species. Mol Immunol. 2008;45(14):3723–30. Epub 2008/07/11. 10.1016/j.molimm.2008.06.002 18614236

[21] Zou J, Mercier C, Koussounadis A, Secombes C. Discovery of multiple beta-defensin like homologues in teleost fish. Mol Immunol. 2007;44(4):638–47. Epub 2006/03/17. 10.1016/j.molimm.2006.01.012 16540171

[22] Wang Q, Bao B, Wang Y, Peatman E, Liu Z. Characterization of a NK-lysin antimicrobial peptide gene from channel catfish. Fish Shellfish Immunol. 2006;20(3):419–26. Epub 2005/07/12. 10.1016/j.fsi.2005.05.005 16005642

[23] Bao B, Peatman E, Li P, He C, Liu Z. Catfish hepcidin gene is expressed in a wide range of tissues and exhibits tissue-specific upregulation after bacterial infection. Dev Comp Immunol. 2005;29(11):939–50. Epub 2005/06/07. 10.1016/j.dci.2005.03.006 15935472

[24] Lauth X, Shike H, Burns JC, Westerman ME, Ostland VE, Carlberg JM, et al Discovery and characterization of two isoforms of moronecidin, a novel antimicrobial peptide from hybrid striped bass. The Journal of biological chemistry. 2002;277(7):5030–9. Epub 2001/12/12. 10.1074/jbc.M109173200 11739390

[25] Chinchar VG, Bryan L, Silphadaung U, Noga E, Wade D, Rollins-Smith L. Inactivation of viruses infecting ectothermic animals by amphibian and piscine antimicrobial peptides. Virology. 2004;323(2):268–75. Epub 2004/06/15. 10.1016/j.virol.2004.02.029 15193922

[26] Silphaduang U, Noga EJ. Peptide antibiotics in mast cells of fish. Nature. 2001;414(6861):268–9. Epub 2001/11/20. 10.1038/35104690 11713517

[27] Buonocore F, Randelli E, Casani D, Picchietti S, Belardinelli MC, de Pascale D, et al A piscidin-like antimicrobial peptide from the icefish Chionodraco hamatus (Perciformes: Channichthyidae): molecular characterization, localization and bactericidal activity. Fish Shellfish Immunol. 2012;33(5):1183–91. Epub 2012/09/18. 10.1016/j.fsi.2012.09.005 22982327

[28] Gautier R, Douguet D, Antonny B, Drin G. HELIQUEST: a web server to screen sequences with specific alpha-helical properties. Bioinformatics. 2008;24(18):2101–2. Epub 2008/07/30. 10.1093/bioinformatics/btn392 18662927

[29] Tamura K, Peterson D, Peterson N, Stecher G, Nei M, Kumar S. MEGA5: molecular evolutionary genetics analysis using maximum likelihood, evolutionary distance, and maximum parsimony methods. Molecular biology and evolution. 2011;28(10):2731–9. 10.1093/molbev/msr121 21546353 PMC3203626

[30] Otvos L, Cudic M. Broth microdilution antibacterial assay of peptides Peptide Characterization and Application Protocols: Springer; 2007 p. 309–20. 10.1007/978-1-59745-430-8_12 18604952

[31] Lin HJ, Huang TC, Muthusamy S, Lee JF, Duann YF, Lin CH. Piscidin-1, an antimicrobial peptide from fish (hybrid striped bass morone saxatilis x M. chrysops), induces apoptotic and necrotic activity in HT1080 cells. Zoolog Sci. 2012;29(5):327–32. Epub 2012/05/09. 10.2108/zsj.29.327 22559967

[32] Clarke A, Crame JA, Stromberg J-O, Barker P. The Southern Ocean benthic fauna and climate change: a historical perspective [and discussion]. Philosophical Transactions of the Royal Society of London B: Biological Sciences. 1992;338(1285):299–309.

[33] Lee YM, Hwang CY, Lee I, Jung YJ, Cho Y, Baek K, et al Lacinutrix jangbogonensis sp. nov., a psychrophilic bacterium isolated from Antarctic marine sediment and emended description of the genus Lacinutrix. Antonie Van Leeuwenhoek. 2014;106(3):527–33. Epub 2014/07/21. 10.1007/s10482-014-0221-5 25038886

[34] Kim SJ, Shin SC, Hong SG, Lee YM, Choi I-G, Park H. Genome sequence of a novel member of the genus Psychrobacter isolated from Antarctic soil. Journal of bacteriology. 2012;194(9):2403–. 10.1128/JB.00234-12 22493207 PMC3347060

[35] Tam JP, Lu YA, Yang JL. Correlations of cationic charges with salt sensitivity and microbial specificity of cystine-stabilized beta -strand antimicrobial peptides. The Journal of biological chemistry. 2002;277(52):50450–6. Epub 2002/10/26. 10.1074/jbc.M208429200 12399464

[36] Masso-Silva JA, Diamond G. Antimicrobial peptides from fish. Pharmaceuticals (Basel). 2014;7(3):265–310. 24594555 10.3390/ph7030265 PMC3978493

[37] Deslouches B, Islam K, Craigo JK, Paranjape SM, Montelaro RC, Mietzner TA. Activity of the de novo engineered antimicrobial peptide WLBU2 against Pseudomonas aeruginosa in human serum and whole blood: implications for systemic applications. Antimicrob Agents Chemother. 2005;49(8):3208–16. Epub 2005/07/29. PubMed Central PMCID: PMC1196285. 10.1128/AAC.49.8.3208-3216.2005 16048927 PMC1196285

